# Metformin and Cancer Glucose Metabolism: At the Bench or at the Bedside?

**DOI:** 10.3390/biom11081231

**Published:** 2021-08-18

**Authors:** Cecilia Marini, Vanessa Cossu, Matteo Bauckneht, Francesco Lanfranchi, Stefano Raffa, Anna Maria Orengo, Silvia Ravera, Silvia Bruno, Gianmario Sambuceti

**Affiliations:** 1 CNR Institute of Molecular Bioimaging and Physiology (IBFM), 20054 Milan, Italy; Sambuceti@unige.it; 2 IRCCS Ospedale Policlinico San Martino, 16132 Genoa, Italy; matteo.bauckneht@hsanmartino.it (M.B.); annamaria.orengo@hsanmartino.it (A.M.O.); 3 Department of Health Sciences, University of Genoa, 16132 Genoa, Italy; vane.6291@gmail.com (V.C.); dr.francescolanfranchi@gmail.com (F.L.); stefanoraffa@live.com (S.R.); 4 Department of Experimental Medicine, Human Anatomy, University of Genoa, 16132 Genoa, Italy; silvia.ravera@unige.it (S.R.); silvia.bruno@unige.it (S.B.)

**Keywords:** metformin, glucose consumption, FDG PET/CT imaging, endoplasmic reticulum, tumor metabolism, cancer therapy

## Abstract

Several studies reported that metformin, the most widely used drug for type 2 diabetes, might affect cancer aggressiveness. The biguanide seems to directly impair cancer energy asset, with the consequent phosphorylation of AMP-activated protein kinase (AMPK) inhibiting cell proliferation and tumor growth. This action is most often attributed to a well-documented blockage of oxidative phosphorylation (OXPHOS) caused by a direct interference of metformin on Complex I function. Nevertheless, several other pleiotropic actions seem to contribute to the anticancer potential of this biguanide. In particular, in vitro and in vivo experimental studies recently documented that metformin selectively inhibits the uptake of 2-[18F]-Fluoro-2-Deoxy-D-Glucose (FDG), via an impaired catalytic function of the enzyme hexose-6P-dehydrogenase (H6PD). H6PD triggers a still largely uncharacterized pentose-phosphate pathway (PPP) within the endoplasmic reticulum (ER) that has been found to play a pivotal role in feeding the NADPH reductive power for both cellular proliferation and antioxidant responses. Regardless of its exploitability in the clinical setting, this metformin action might configure the ER metabolism as a potential target for innovative therapeutic strategies in patients with solid cancers and potentially modifies the current interpretative model of FDG uptake, attributing PET/CT capability to predict cancer aggressiveness to the activation of H6PD catalytic function.

## 1. Introduction

Since the 1950s, the biguanide metformin has been the most widely used antihyperglycemic drug to treat patients with type 2 diabetes. This drug exerts its effects by reducing hepatic gluconeogenesis [1,2] and by increasing insulin sensitivity as well as glucose consumption of peripheral tissues [3]. Besides this classical indication, a wide literature proposed a potential efficacy of this biguanide in anticancer therapy, either alone or in combination with other approaches [4]. The mechanisms underlying this action extend beyond the antihyperglycemic action and possibly identify an anticancer potential of metformin in nondiabetic patients.

**Figure 1:**
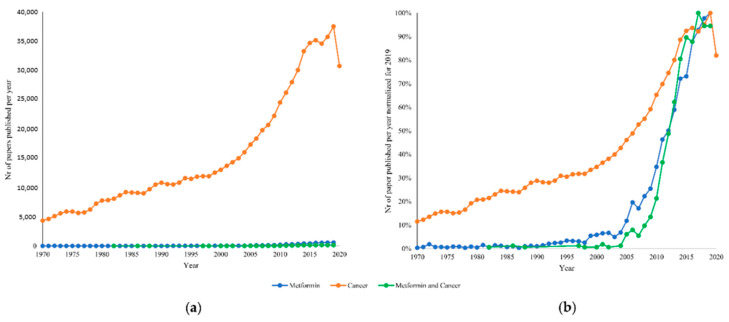
(**a**) The number (nr) of papers on experimental animals, published each year since 1970 and up to 2020, containing the terms “metformin”, “cancer”, and “metformin and cancer”. (**b**) The same data displayed as the ratio between the nr of papers published in each year divided by the corresponding feature in 2019. The steep increase in experimental studies dealing with metformin was associated with the interest in the anticancer potential of this drug.

As an obvious consequence, these expectations increased the interest in metformin up to contaminate the field of basic research activity, as documented by the PubMed database reported in Figure 1 that shows how (and when) the number of studies on experimental animals and containing the terms “metformin” grew simultaneously with the expansion of “non-clinical” studies containing the terms “metformin and cancer”.

Metformin anticancer potential has been attributed to the interference with key pathways, described by several excellent reviews [5,6,7], and related to energy metabolism, proliferating activity, and migratory potential of cancer cells as well as to inflammatory and immune host reaction.

Nevertheless, this wide literature did not solve yet the debate about the real potential of metformin in cancer therapy, since concerns exist about its effectiveness in the different cancer types preventing any possible definition about its clinical indications in the clinical practice.

Despite this uncertainty, the evident metformin effect in several experimental models of solid tumors indicates that the approach to energy metabolism might represent a new, potentially effective approach to neoplastic patients [8,9]. This review aims thus to provide a detailed and, under some aspects, challenging description of the mechanism underlying metformin interference on energy metabolism and glucose consumption as possible clues to develop innovative approaches for metabolic targeting in cancer.

## 2. Effect of Metformin on Glucose Metabolism in Cancer

At the molecular level, the most recognized target of metformin action is the Complex I of the respiratory chain [10,11]. Indeed, the positively charged biguanide can cross both cell and mitochondrial membranes, leading to a selective accumulation into the mitochondrial matrix, where metformin concentration can reach values up to 1000-fold higher than in extracellular medium [12]. Drug direct inhibitory effect on Complex 1 has been confirmed by the observation of a decreased oxygen consumption rate combined with a decrease in mitochondrial membrane potential in permeabilized cells and isolated mitochondria [13]. Nevertheless, the mechanism of action of metformin probably extends to other cell structures. Indeed, this biguanide has been found to also modify glucose metabolism of erythrocytes that are devoid of these organelles, possibly suggesting drug-induced change in cell membrane fluidity [14].

The inhibition of OXPHOS inevitably results in a reduced production rate of ATP and thus in an evident impairment of cell energy asset, the consequent decrease in the ATP/AMP ratio eventually activating energy sensor mechanisms shared by virtually all eukaryotic cells and able to enhance catabolic processes while switching off the anabolic ones in order to restore the cell. Among these pathways, a relevant role is played by the activation of the tumor suppressor gene liver kinase B1 (LKB1) [15] that eventually promotes the phosphorylation of AMPK to its active form P-AMPK [16,17].

As a further consequence of OXPHOS inhibition, metformin has been found to induce an increase in the lactate/pyruvate ratio, suggesting an impaired cell capability to re-oxidize the cytoplasmic NADH, possibly caused by a direct inhibition of the mitochondrial glycerophosphate dehydrogenase [18].

Besides these effects and the consequent deceleration in gluconeogenesis, the impaired energy asset implies a switch-off of G6P-phosphatase expression through mechanisms at least partially independent of AMPK activation and most likely related to the inhibitory effect by the direct AMP-dependent inhibition of adenylate cyclase [19]. Accordingly, the energetic impairment caused by therapeutic metformin doses impairs the function of serum glucose buffering organs (liver, kidneys, and gut), slowing down their release of free glucose into the bloodstream, thus explaining the antihyperglycemic action of this biguanide [20].

In cancer cells, the OXPHOS inhibition activates the LKB1–AMPK pathway, thus inhibiting the Raptor–mTOR complex (mammalian TOR complex 1 (mTORC1)) and the tuberous sclerosis complex 2 (TSC2) [21]. The effect of this reaction is most active on GTPase-activating protein that inactivates the small GTP-binding protein Ras Homolog Enriched in Brain (RHEB), eventually decreasing mRNA translation and ribosome biogenesis [22]. This series of events combines with the P-AMPK capability to inhibit cyclin d1 protein expression, preventing cell entry into the S-phase of cell cycle with the final result of an inhibited proliferation rate, although not associated with the induction of apoptosis [23,24,25,26]. Further, the capability of metformin to activate AMPK phosphorylation has been found to extend to prevent the genetic and epigenetic alterations featuring many cancer phenotypes. Indeed, Wu and coworkers recently reported a P-AMPK-dependent stabilization of the tumor suppressor ten-eleven translocation protein (TET2) in xenograft models of tumors implanted in diabetic mice [27].

According to these considerations, the energetic stress directly caused by metformin might provide a favorable therapeutic index in a sizable number of cancers, mostly in those characterized by an overexpression of mTOR. The faster progression and poorer prognosis of these lesions might indeed be most sensitive to the downregulation of mTOR transcription factors and the reduction of insulin-like growth factors caused by this treatment [28].

Finally, the response of whole-body metabolic pattern to metformin intake indirectly modifies the systemic signaling to cancer lesion. Indeed, the antihyperglycemic drug action eventually results in decreased plasma concentrations of growth-supporting hormones insulin-like growth factor 1 (IGF1) and insulin [29].

## 3. Metformin and Cancer Glucose Metabolism by Direct Inhibition of Hexokinase-II

Under several aspects, the severe consequences of metformin-induced OXPHOS inhibition on cancer cell biology might appear somewhat paradoxical. Indeed, since the seminal studies by Otto Warburg, cancer metabolism is known to display a high glycolytic flux and lactate release even under exposure to high oxygen tension (aerobic glycolysis) [30,31]. Like all glucose degradation pathways, glycolysis implies the availability of G6P derived from the reaction catalyzed by hexokinases (HKs). In all mammalians, HKs exist in four isoforms that differ in catalytic and regulatory properties as well as subcellular localization. Differently from the neuronal HK3 and the liver-pancreas HK4, HK1 and HK2 are ubiquitously expressed. Both isoforms are tightly bound with the outer mitochondrial membrane [32] and are thus empowered with a privileged access to ATP produced by the OXPHOS. In the outer mitochondrial membrane, in fact, both isoforms interact with the permeability transition pore, which includes the voltage-dependent anion channel 1 (VDAC1) responsible for ATP flux to the cytoplasm. This physical association facilitates glucose phosphorylation, protects cell from apoptosis [33] and its enhanced expression pattern predicts a poor prognosis in cancer patients, playing a key role in cancer growth and survival [34].

In silico studies indicate that metformin interacts with HK2 pocket for glucose, selectively inhibiting the catalytic function of this enzyme by preventing the connection between the glucose itself and ATP already at concentrations around 100 µM. This inhibition is paralleled by a detachment of HK2 from the mitochondrion. In the same way as G6P, the inhibitory effect of metformin on HK2 is inversely related to the ATP concentration. Thus, ATP depletion caused by the respiratory blockade coupled with the enzyme detachment from the mitochondrial outer membrane eventually results in a marked inhibition of HK2 activity further decreasing the availability of G6P [35].

Altogether, thus, these data seem to suggest that the slow-down in cancer growth cause by metformin might represent the consequence of respiratory inhibition combined with a decreased fueling of G6P for both glycolysis and PPP.

## 4. Moving from the Bench to the Bedside: What Is Anticancer Potential of Metformin?

The wealth of studies and observations reported above indicate a powerful anticancer potential for metformin largely independent of its antihyperglycemic action. A similar consideration also applies to experimental models of tumor-bearing mice, in which metformin has been found to slow down cancer progression and to improve the effectiveness of both radiotherapy [36] and chemotherapy [37]. Nevertheless, its clinical use in cancer prevention or therapy has not been conclusively defined.

A major problem in this setting relies on the dosage schedule adopted in basic research studies. In vitro, metformin metabolic effect has been reported for very high drug concentrations ranging from 1 to 10 mM [35,38,39,40,41]. Similarly, in experimental mice, metformin was administered at dosages often greater than 500 mg/kg daily. This dose regimen would roughly correspond to 40 g per day in a patient with 80 kg body weight, that is, 20 times higher than the maximal dose usable for clinical purposes. Such a dose scheduling is obviously not achievable in the clinical practice, and thus the metformin action documented in the experimental setting cannot be extended to its possible effect in patients.

Nevertheless, this caveat might be probably less stringent in cancers of the gastro-enteric or the urinary tracts. Indeed, both locations expose the lesion to relatively high drug concentrations due to its oral administration and its urinary excretion. Indeed, [^11^C]-labeled metformin has been found to be highly concentrated in gut, liver, kidneys and bladder [42], far beyond the plasma level [43,44]. This selective exposure most likely explains the great attention that has been paid to the use of this biguanide in this setting in colon-rectal cancer that encompassed more than 20 clinical studies [45] with respect to the 11 reports focused on breast [46], 18 on the lung [47], and nine on prostate tumors [48]. From a pharmacokinetic point of view, the liver exposure to orally administered drugs relies on the fact that about 80% of the blood supply to this organ comes from the venous outflow from gut through the portal vein. Nevertheless, it might be less applicable to the vascular structure of liver cancer lesions (either primary or repetitive) whose supply is most largely dependent upon the hepatic artery.

Accordingly, at present, the therapeutic potential of metformin in cancer patients still has to be fully clarified. Although a relatively wide literature suggests a potential role for this drug in cancer treatment and prevention, still we miss the definitive evidence for its effectiveness provided by prospective double-blind trials. Even more, we still miss a thoughtful definition of which patients can be considered candidates for this drug as well as which therapeutic combinations might offer a measurable benefit.

## 5. Moving from the Bench to the Bedside: Does Metformin Hamper the Clinical Accuracy of FDG Imaging?

According to the quoted experimental evidence, the OXPHOS impairment and the consequent energetic imbalance induced by metformin should result in an accelerated glycolytic flux despite the direct inhibition of HKII activity. This concept has been confirmed by several studies [39,49] that documented an increased lactate release in cultured cancer cells exposed to the biguanide. This metabolic response might thus improve the diagnostic accuracy of FDG imaging that so far represents a clinical standard in most patients with solid cancer.

Current interpretation of FDG uptake derives from the seminal work by Sokoloff et al. [50], which described the tight and local connection between glucose consumption and ^14^C-2-deoxyglucose (2DG) retention in the brain. Phelps and coworkers extended the kinetic model to FDG to humans, allowing the subsequent method optimization for patients with different disorders [51]. According to this model, FDG uptake competes with glucose for both transmembrane-facilitated transport (through GLUT) and entrapment through the HK-catalyzed phosphorylation [52,53]. However, FDG-6-phosphate (FDG6P) accumulates in the cytosol as false substrate for gatekeepers of either glycolysis (G6P-isomerase) or pentose-phosphate pathway (PPP) (G6P-dehydrogenase, G6PD). Consequently, FDG uptake represents an indirect, though robust, index of overall glucose consumption. In turn, the direct link between this metabolic feature and proliferating activity accounts for the validity of FDG uptake as an index of cancer aggressiveness [54].

This model is almost universally accepted. However, it does not fit with some basic observations. On the clinical ground, FDG uptake is low in prostatic, urothelial, and neuroendocrine cancer cell lines despite a high glucose consumption [38,39]. On the experimental ground, previous studies have shown that more than half of the radioactivity is retained within the cell in a different form with respect to FDG6P [55]. Finally, on a theoretical level, the irreversible accumulation of the tracer in cancer does not fit with the high and ubiquitous expression of the enzyme G6P-phosphatase, whose compartmentalization within the ER requires the access to FDG6P to carry out its catalytic function [56]. All tissues and solid cancers display a measurable, though usually very slow, radioactivity loss, indicating either a slow transport rate through the ER membrane or the presence of an enzymatic system competing with G6Pase for FDG6P hydrolysis.

Based on these considerations, we tried to verify whether metformin does increase FDG uptake in cultured cells. However, cell cultures incubated with the biguanide showed a marked and dose-dependent decrease in tracer retention [38] facing a marked increase in glycolytic flux. This mismatch was reproduced in a large panel of cell lines representative of different forms of cancer [38,41] and in neurons [40].

Unexpectedly, these evaluations documented that the divergent effect of metformin on FDG uptake and glucose consumption is largely explained by the drug effect on the catalytic function of an autosomic enzyme located in the ER: H6PD [38]. Although scarcely characterized, H6PD appears relevant in FDG retention mostly because it recognizes many phosphorylated and free hexoses as substrates to trigger a PPP selectively located within the ER lumen [57,58].

In our evaluation, metformin did not alter the expression of both H6PD and G6PD. By contrast, the decreased FDG uptake was coherent with a selective impairment of H6PD catalytic function that almost halved cell capability to dehydrogenate 2DG6P without affecting G6PD activity measured by G6P dehydrogenation rate [38]. Similarly, the same response of tracer retention and lactate release was reproduced by H6PD silencing with short interfering RNA (siRNA) [38].

The connection between FDG uptake and H6PD activity was further confirmed by confocal microscopy in both cancer and normal cells: in untreated cells, the fluorescent FDG analogue (2-[N-(7-nitrobenz-2-ossi-1,3-diazol-4-yl)amino]-2-desossiglucose, 2-NBDG) co-localized with the ER membrane, identified by the fluorescent probe glibenclamide. This distribution pattern was markedly and comparably altered by both high metformin doses and H6PD silencing [38,40,41].

Altogether, these observations would configure H6PD function as a primary determinant of the metabolic adaptation to cell phenotype and differentiation. This concept apparently disagrees with the very low expression of this enzyme, whose level was 300 times lower than its cytosolic counterpart G6PD, in our preliminary proteomic analysis. Nevertheless, Cossu et al. used nuclear magnetic resonance spectroscopy to verify the metabolic response to the inhibition of H6PD or G6PD expression with siRNA in human cancer cell lines [41]. Silencing either enzyme comparably decreased PPP intermediates and NADPH/NADP ratio and similarly enhanced the cell content of reactive oxygen species. These data thus suggested that H6PD processes comparable G6P amounts with respect to G6PD. Similarly, they also indicate that the ER and cytosolic PPPs act on different G6P pools without any interference on the reciprocal activity.

Based on these considerations, we hypothesized that FDG uptake might reflect the degree of activation of H6PD-triggered ER-PPP also in normal tissues. Actually, FDG uptake in normal mouse brain was decreased by the prolonged treatment with high doses of metformin [40]. This effect was reproduced ex vivo and, again, it mismatched the marked increase in glucose consumption induced by the drug-related inhibition of respiratory activity.

**Figure 2:**
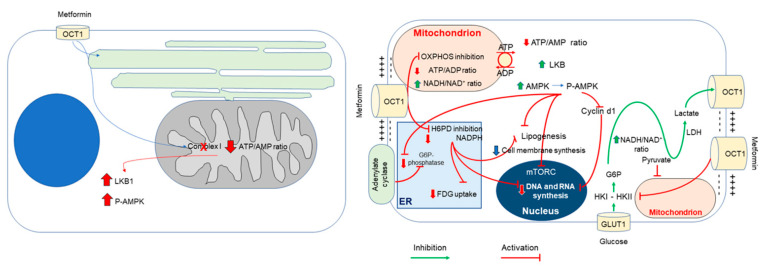
Schematic representation of metformin effects on intracellular glucose metabolism.

Classical models of cell biochemistry define PPP as the main pathway able to preserve the NADPH levels [59,60]. The reductive power of this cofactor is fundamental to support two basic biological processes: on one side, it plays a key role in the reductive biosynthesis needed by cellular proliferation; on the other hand, it is the main reductive agent used by glutathione-dependent antioxidant responses. As a matter of fact, synthetizing cell membrane fatty acids asks for 35 times more glucose equivalents to feed NADPH-derived electrons than for the needed ATP moieties [31]. According to these observations, the ER pool of this co-factor seems thus particularly relevant for cancer proliferation, while the selective interference of metformin on ER PPP might represent a potential target for innovative metabolic approaches to cancer therapy (Figure 2).

In the clinical setting, the relevance of these observations mostly applies to the high number of patients with type 2 diabetes submitted to PET/CT imaging. Indeed, the competition between FDG and unlabeled glucose for cell entry possibly leads to an underestimation of lesion glucose consumption when scanning is performed in the presence of hyperglycemia. This concept has been accepted by current procedural guidelines that discourage FDG imaging in patients with fasting glucose levels up to 200 mg/dL [61]. To avoid this limitation, these subjects are submitted to imaging under active treatment with metformin that might decrease tracer uptake.

Despite the wide use of the biguanide in this setting, this potential pitfall and its interference with the diagnostic accuracy of PET/CT imaging has been scarcely addressed in the literature. Actually, we observed a slight reduction of the avidity for FDG in cancers grown in chronically and mildly hyperglycemic mice, previously treated with streptozotocin. Nevertheless, this response was paralleled by a loss of sensitivity to metformin, which involved both FDG accumulation rate and lesion growth [62]. On one side, this response was coherent with the notion that hyperglycemia can inhibit metformin capability to activate AMPK phosphorylation [63]. More importantly, it was explained by the behavior of H6PD whose catalytic function was directly and linearly correlated with tracer uptake in all evaluated lesions. This response was matched by the G6PD and paralleled the expected increase in redox stress observed in hyperglycemic mice, again suggesting the relevance of PPP activation as a primary determinant of FDG activation.

As a final consideration, however, a large uncertainty still exists about the potential interference of metformin treatment in PET diagnosis of colorectal cancer. Indeed, in this setting, the drug-induced inhibition of FDG uptake combines with a marked increase in tracer retention of normal colonic enterocytes, both in patients and in experimental models [64,65]. As a consequence, the optimal procedure for patient preparation in this setting is still a matter of debate about the need for drug discontinuation as well as about the duration of this treatment change before the exam [66,67].

## 6. Conclusions

Although not conclusively duplicated in the clinical setting, the capability of metformin to inhibit proliferating activity and migratory potential of cancer cells has been documented by a wealth of studies both in vitro and in experimental animals. In agreement with the mechanism underlying the drug antihyperglycemic action, this pharmacological effect involves the biguanide capability to bind the respiratory Complex I and thus to inhibit the OXPHOS. The resulting impairment in cell energy asset activates AMPK phosphorylation, switching off the reductive syntheses needed for cancer growth. Nevertheless, besides this well-documented action, metformin anticancer potential extends to involve its capability to slow down the rate of a specific PPP located within the ER, whose activity has been found to be represented in many solid cancers.

The reactions sequence, the controlling mechanisms, the exchange with cytosol metabolites and the ultimate role of this reticular pathway are still largely undefined. Yet, its role in feeding the NADPH reductive power for the synthesis of both nucleic acids and cell membranes has been found to be unexpectedly relevant and might thus represent a new, largely unexplored, target in anticancer therapy. Thus, the investigation on metformin effect so far provided a new therapeutic approach to oncological patients. The missing adherence of clinical data with experimental findings might probably reflect several explanations and mostly the large difference in the used dosages. Nevertheless, obtained data indicate the need for studying the therapeutic potential of other compounds targeting the ER metabolism.
